# Human Adipose-Derived Stromal/Stem Cell Culture and Analysis Methods for Adipose Tissue Modeling In Vitro: A Systematic Review

**DOI:** 10.3390/cells10061378

**Published:** 2021-06-03

**Authors:** Peyton Gibler, Jeffrey Gimble, Katie Hamel, Emma Rogers, Michael Henderson, Xiying Wu, Spencer Olesky, Trivia Frazier

**Affiliations:** 1 Obatala Sciences Inc., New Orleans, LA 70148, USA; peyton.gibler@obatalasciences.com (P.G.); katie.hamel@obatalasciences.com (K.H.); emma.rogers@obatalasciences.com (E.R.); michael.henderson@obatalasciences.com (M.H.); xiying.wu@obatalasciences.com (X.W.); spencer.olesky@obatalasciences.com (S.O.); trivia.frazier@obatalasciences.com (T.F.); 2 Department of Structural and Cell Biology, Tulane University School of Medicine, New Orleans, LA 70112, USA; 3 Department of Medicine, Tulane University School of Medicine, New Orleans, LA 70112, USA; 4 Department of Surgery, Tulane University School of Medicine, New Orleans, LA 70112, USA

**Keywords:** adipose-derived stromal/stem cells, culture methods, 3-dimensional, microphysiological system

## Abstract

Human adipose-derived stromal/stem cells (hASC) are widely used for in vitro modeling of physiologically relevant human adipose tissue. These models are useful for the development of tissue constructs for soft tissue regeneration and 3-dimensional (3D) microphysiological systems (MPS) for drug discovery. In this systematic review, we report on the current state of hASC culture and assessment methods for adipose tissue engineering using 3D MPS. Our search efforts resulted in the identification of 184 independent records, of which 27 were determined to be most relevant to the goals of the present review. Our results demonstrate a lack of consensus on methods for hASC culture and assessment for the production of physiologically relevant in vitro models of human adipose tissue. Few studies have assessed the impact of different 3D culture conditions on hASC adipogenesis. Additionally, there has been a limited use of assays for characterizing the functionality of adipose tissue in vitro. Results from this study suggest the need for more standardized culture methods and further analysis on in vitro tissue functionality. These will be necessary to validate the utility of 3D MPS as an in vitro model to reduce, refine, and replace in vivo experiments in the drug discovery regulatory process.

## 1. Introduction

Stem cells are widely used for in vitro tissue engineering due to their ability to self-renew and differentiate into multiple cell lineages [1]. In the beginning of the 21st century, human adipose-derived stromal/stem cells (hASC) emerged as a source of stem cells capable of differentiating along several lineage pathways including adipogenic, chondrogenic, osteogenic, endothelial, cardiac, and neural [2]. hASC are an abundant source of cells that can be isolated from subcutaneous adipose tissue through minimally invasive procedures. Their abundance and relative ease of isolation make hASC advantageous over other stem cell types, such as bone marrow-derived stem cells that require more invasive procedures for acquisition [3].

Adipose tissue recently emerged as a research area of great interest due to its endocrine activity and role in disease conditions such as obesity [2,4]. According to the Centers for Disease Control, the prevalence of obesity in adults has increased from 30.5% in 1999–2000 to 42.2% in 2017–2018 (https://www.cdc.gov/obesity/data, accessed on 10 February 2021). Obesity is associated with several comorbidities including type 2 diabetes, cardiovascular disease, and metabolic syndrome; therefore, an understanding of the mechanisms driving obesity and discovery of prevention methods are of great interest [4].

In vitro models of adipose tissue can be constructed using hASC cultured in adipogenic inductive medium to study adipose tissue biology and pathophysiology. Many in vitro studies involve culturing cells in a 2-dimensional (2D) format; however, this technique fails to recapitulate the native 3-dimensional (3D) microenvironment. Therefore, 3D cell culture methods have been developed to better model the structure and function of human tissue as compared to their 2D counterparts. 3D cell culture mechanisms involve seeding cells into a scaffold or allowing them to self-assemble into cell aggregations called spheroids in a scaffold-free environment.

**Figure 1:**
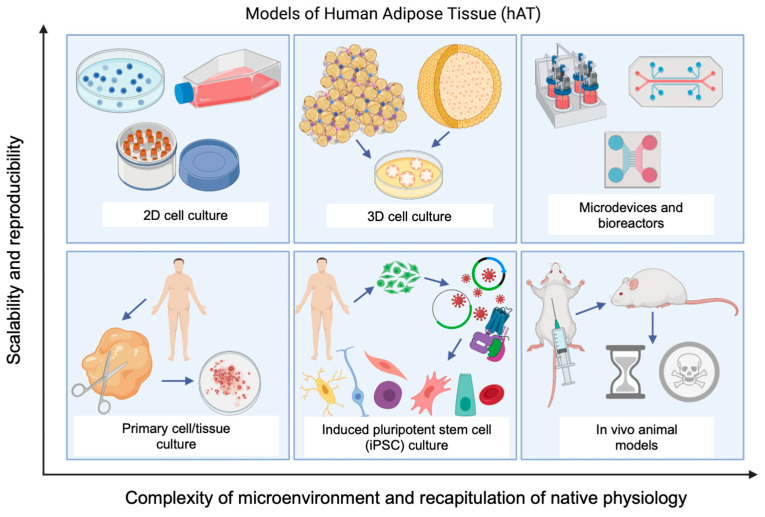
Diagram demonstrating the scalability, reproducibility, complexity, and physiological relevance of available human adipose tissue models. Image created with BioRender.com.

Scaffold and scaffold-free culture conditions can be employed to engineer constructs for implantation and soft tissue regeneration or to develop adipose tissue microphysiological systems (MPS). MPS are defined as 3D, multicellular, tissue-engineered organ constructs developed using human cells [5]. MPS have been developed out of a need identified by the U.S. Food and Drug Administration (FDA) to develop tools for enhanced prediction of toxicological risk of medical products entering the market [6]. MPS are designed for pathophysiological and pharmacological research on human tissue in vitro. MPS aim to reduce, refine, and eventually replace animal models that are costly and time-consuming by creating a more scalable and reproducible platform for discovery (Figure 1). In vitro models of human adipose tissue will serve to further scientific understanding of adipose tissue biology and dysfunction associated with disease conditions such as obesity. Additionally, healthy and diseased MPS platforms will facilitate more efficient drug discovery processes by modeling human responses to new pharmacological agents.

The purpose of this review is to evaluate emerging methods of culturing and assessing hASC in vitro for the development of engineered adipose tissue constructs. The identification of the most physiologically relevant culture and characterization methods for engineering constructs that recapitulate native human adipose tissue will support efforts in soft tissue regeneration and in vitro modeling for drug discovery.

## 2. Methods

We conducted a systematic review by searching PubMed using 7 subsets of keywords as defined in Table 1. Additional articles of known relevance acquired through independent search efforts were also included to ensure a thorough review of the published literature. The articles collected for this review focused on literature published between 2000 and 2021.

Duplicate articles identified using different subsets of keywords were removed from the original search results. Review articles were excluded from analysis as were articles that did not utilize human adipose-derived stromal/stem cells (hASC) or stromal vascular fraction (SVF) cells. As a note, SVF cells represent all other cell subtypes besides the mature adipocytes in adipose tissue, and contain endothelial progenitors, fibroblasts, lymphocytes, monocyte/macrophages, pericytes, pre-adipocytes, and stromal/stem cells, among others [7]. This collective population of cells is gaining increasing attention as we gain more insight into the contribution of adipose tissue cellular composition to overall structure and depot functionality, neuroendocrine function, viral response, and homeostasis regulation. Finally, articles were excluded if the full text could not be obtained beyond attempts of interlibrary sourcing within the US.

## 3. Results and Discussion

**Figure 2:**
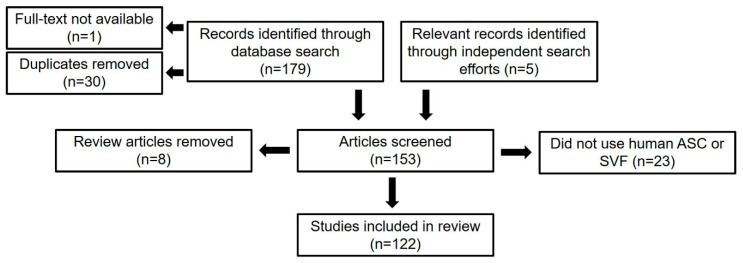
Flow diagram of literature selection process (n = number of articles).

Our database search and independent acquisition of relevant articles resulted in the identification of 184 records. After 30 duplicates were removed as well as one article for which the full text could not be obtained, 153 independent articles were screened further based on the abovementioned exclusion criteria. Thirty-one articles were excluded in this screening process, leaving 122 records to be analyzed in the systematic review (Figure 2). Results from this review are presented as either the percentage of appearances in the literature or the percentage of studies that utilized a specific method.

### 3.1. Differentiation Pathways

**Figure 3:**
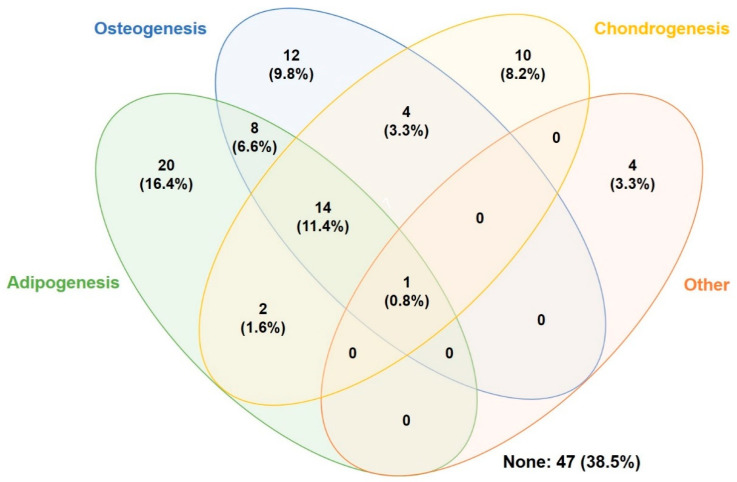
Venn diagram of differentiation pathways studied. Results given as percentage out of 122 total papers.

Human adipose-derived stromal/stem cells are capable of differentiation along multiple lineage pathways. As such, this database search identified studies on a variety of differentiation pathways. The most common hASC differentiation pathways studied were adipogenic (36.9%) [4,5,8,9,10,11,12,13,14,15,16,17,18,19,20,21,22,23,24,25,26,27,28,29,30,31,32,33,34,35,36,37,38,39,40,41,42,43,44,45,46,47,48,49,50], osteogenic (32.0%) [4,29,30,31,32,33,34,35,36,37,38,39,40,41,42,43,44,45,46,47,48,49,50,51,52,53,54,55,56,57,58,59,60,61,62,63,64,65,66], and chondrogenic (25.4%) [27,28,36,37,38,39,40,41,42,43,44,45,46,47,48,49,50,63,64,65,66,67,68,69,70,71,72,73,74,75,76]. Several articles studied a different type of differentiation, which was designated other (4.1%) [50,77,78,79,80], while 38.5% [81,82,83,84,85,86,87,88,89,90,91,92,93,94,95,96,97,98,99,100,101,102,103,104,105,106,107,108,109,110,111,112,113,114,115,116,117,118,119,120,121,122,123,124,125,126] of articles reviewed did not study any kind of differentiation (Figure 3).

**Figure 4:**
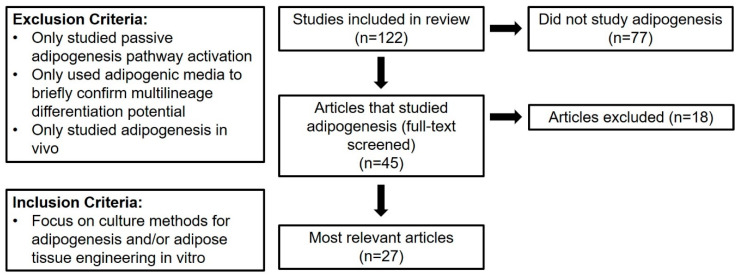
Flow diagram of selection process to identify articles that are most relevant to the present review. These articles focus on intentional human adipose-derived stromal/stem cells (hASC) adipogenesis and adipose tissue engineering in vitro.

Articles that assessed adipogenesis were divided further according to the flow chart in Figure 4. The full texts of the 45 articles that studied adipogenesis were screened using the exclusion criteria outlined in Figure 4. Records were excluded if they exclusively studied passive adipogenesis pathway activation, if they only used adipogenic media to confirm multilineage differentiation potential, or if they exclusively studied adipogenesis in vivo. Many articles assessed the passive activation of adipogenic pathways to ensure that their culture conditions did not encourage spontaneous adipogenic differentiation. Additionally, many articles did not focus on adipogenesis but only used adipogenic media to demonstrate the multilineage differentiation potential of hASC. These articles were filtered to ensure that the data presented in this review reflect methods for intentional hASC adipogenesis and adipose tissue engineering in vitro. Finally, none of the screened articles exclusively studied 2D culture; therefore, all articles included in this analysis report 3D culture methods. Data and analysis hereafter exclusively refer to the 27 articles [4,5,8,9,10,11,12,13,15,16,17,19,20,21,22,23,24,25,26,28,30,31,33,38,44,45,48] identified to be most relevant to the central goal of this systematic review.

Out of the 27 relevant articles, 26 articles used adipogenic medium to differentiate hASC into adipocytes in vitro. The studies reviewed used either commercial (40%) or custom-made medium (60%). Table 2 highlights the prevalence of adipogenic medium components used in custom-made medium. The variability of components used as well as their respective concentrations indicates a lack of consensus in the literature regarding the most effective methods for adipogenic induction of hASC.

### 3.2. Adipogenic Differentiation Analysis Techniques

#### 3.2.1. Microscopy Techniques

**Figure 5:**
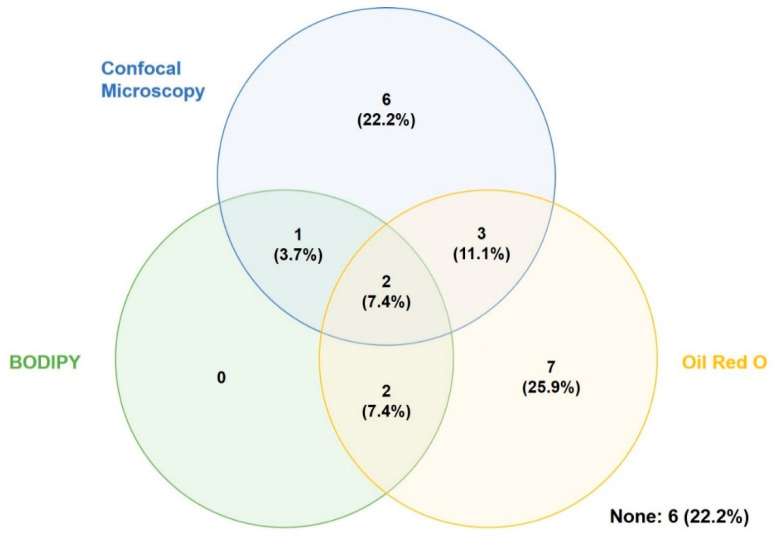
Venn diagram of microscopy techniques used to evaluate hASC in vitro. Results given as a percentage out of the 27 relevant articles.

In vivo, mature adipocytes often have a complex three-dimensional structure that is characterized by a single large (unilocular) lipid droplet along with other cellular components contained within a thin cytoplasm. Immature adipocytes are described as multilocular as they contain multiple small lipid droplets. Throughout maturation, these small lipid droplets aggregate together to form the aforementioned unilocular droplet [127]. This maturation process can be observed visually and described quantitatively through various microscopy techniques and is referred to as a signet ring structure on the basis of appearance (Figure 5).

It can be challenging to image adipocytes using light microscopy because of their high lipid content which causes autofluorescence that is enhanced by light diffraction. Confocal imaging, used in combination with the introduction of fluorescently-tagged lipophilic dyes, is a useful alternative as it operates in a narrow plane of focus allowing for the removal of autofluorescence. Additionally, a z-stack of images can be combined to recreate an image of the complex three-dimensional structure of adipocytes [128]. Of articles included in this review, 44.4% used confocal microscopy.

Oil red O (ORO) is a staining solution that accumulates in cellular compartments that are lipid rich; therefore, it can be used to detect the neutral lipid amounts after hASC adipogenesis. ORO can be observed using bright field and fluorescent microscopy [16]. Of articles included in this review, 51.9% used ORO staining to qualitatively observe neutral lipid content; however, only one article included in this review (3.7%) used ORO staining to quantitatively measure adipogenesis by eluting the remaining stain with isopropanol and measuring the absorbance of the solution [129].

Alternatively, BODIPY is a commercially available fluorescent dye that labels lipid droplets and is detected as bright green fluorescence [130]. Of articles reviewed, 18.5% used BODIPY to assess lipid accumulation in their culture conditions.

#### 3.2.2. Snapshot Assays

Analyses associated with a specified single time point or “snapshot” assay are used to quantitatively assess hASC biomarker expression. Snapshot assays confirm the status of cells at a given time point but lack substantial information about their temporal functionality in vitro.

Flow cytometry is a widely used assay for assessing the immunophenotype of mesenchymal stromal/stem cells (MSCs). According to the International Federation for Adipose Therapeutics (IFATS) and Science and the International Society for Cellular Therapy (ISCT), hASC in culture maintain markers similar to other MSCs as they are positive for CD90, CD73, CD105, and CD44 and negative for CD45 and CD31 [25]. Flow cytometry was used by 18.5% of reports included in this review to characterize hASC immunophenotype. The reports uniformly confirmed the positive expression of biomarkers CD90 and CD105 while all other markers varied between publications (Table 3).

Quantitative reverse transcriptase polymerase chain reaction (qRT-PCR) is a quantitative method to assess gene expression pre and post-hASC adipogenesis. Of articles evaluated in this review, 40.7% employed qRT-PCR to assess adipogenesis-related gene expression. The most commonly assessed genes were peroxisome proliferator activated receptor γ (PPAR-γ) (84.6% of articles using qRT-PCR), lipoprotein lipase (LPL) (46.2%), fatty acid binding protein 4 (FABP4) (46.2%), and adiponectin (ADIPOQ) (30.8%). hASC adipogenesis is driven by PPAR-γ expression and characterized by the upregulation of LPL, FABP4, and ADIPOQ [131].

#### 3.2.3. Functional Biochemical Assays

Functional biochemical assays quantify the dynamic functionality of cellular enzymatic activities based on biomarkers at the carbohydrate, lipid, or protein levels. Four functional biochemical assays were routinely employed monitoring triglyceride, lipolysis, glucose uptake, and adipokine secretion. The ASCs’ accumulation of neutral triglycerides with the formation of intracellular lipid droplets was assessed quantitatively based on biochemical detection of triglyceride levels. Following triglyceride lysis into free fatty acids and glycerol, glycerol levels are evaluated based on spectrophotometric detection. Alternatively, the ASC intracellular lipolytic enzyme activity can be monitored following stimulation with exogenous isoproterenol based on release of glycerol within the cellular lysates [11]. Both assays were employed in 14.8% of reviewed reports.

Glucose uptake following activation of the GLUT4 transporter in response to insulin can be monitored based on colorimetric or fluorescent detection of 2-deoxyglucose uptake by ASC. Only a single report in this systematic review (3.7%) reported this outcome. Finally, adipokine assays using antibody-based methods (ELISA or bead-based assays) are used to quantify ASC cytokine secretion activity. While 25.9% of the reports evaluated adipokine secretion, none of the articles characterized the cellular inflammatory response in the context of adipogenesis.

### 3.3. 3D Culture Mechanisms

Three-dimensional stromal/stem cell culture techniques have been developed for the purpose of engineering in vivo-like adipose tissue constructs. Three-dimensional culture mechanisms can be subdivided into either scaffold or scaffold-free culture systems. Likewise, culture conditions can also be described as either static or dynamic. In static culture, cells are placed in non-circulating media that is replaced every few days throughout the culture period. In dynamic culture, media is continuously perfused through the culture construct to model the circulating in vivo environment.

#### 3.3.1. Scaffold Culture

One critical aspect of 3D stem cell culture is developing an in vitro environment that closely mimics native in vivo conditions [11,21,25]. A widely used strategy is to seed stem cells into a scaffold that is designed to recapitulate the native extracellular matrix (ECM). Scaffolds can be composed of biologically derived or synthetic materials. Out of the relevant records reviewed herein, 74% of articles utilized a scaffold material for the culture of hASC. The most common synthetic and biologically derived materials used in the literature are highlighted in Table 4. Only one article (5%) used an entirely synthetic scaffold, while the remaining articles incorporated biologically derived materials. Gelatin was identified as one of the most commonly used scaffold materials as it naturally contains RGD peptides found in many ECM components that promote cell adhesion [13,16,17,21]. Collagen is another commonly used scaffold material as it is the most abundant protein in human adipose tissue ECM and, therefore, closely recapitulates the native tissue environment [10,31]. hASC attachment and adipocyte maturation have been shown to impact scaffold structure [10,16]. Additionally, scaffold material and properties have been shown to impact hASC adipogenesis [16]. Notable articles identified in this review characterized cell-scaffold interactions throughout hASC attachment, proliferation, and differentiation.

Newman et al. developed collagen-elastin-like polypeptide scaffolds for culturing hASC and found that more stiff, dense, and crosslinked scaffolds led to a spheroid morphology whereas less stiff, non-crosslinked scaffolds lead to a spread morphology. The spheroid morphology is more representative of native adipose tissue cell morphology, which may explain why cells seeded on stiff, crosslinked scaffolds experienced enhanced adipogenesis compared to their less-stiff, non-crosslinked counterparts [9].

Clevenger et al. developed three different hydrogels with various RGD peptides (cyclical, linear, and derived from vitronectin) to assess impact on hASC adhesion, proliferation, and differentiation. Their results suggested that the cyclic and vitronectin-derived RGD sequences allowed for robust initial attachment of undifferentiated hASC. Vitronectin-derived RGD maintained cells in a more rounded morphology. This rounded morphology supported increased adipogenesis in hydrogels containing vitronectin-derived RGD compared to other RGD peptides as determined by percentage of cells containing lipid vacuoles as well as lipid vacuole size [8,31].

Despite the work by Newman et al. and Clevenger et al., studies on the interaction between ECM-derived scaffold biomaterials and hASC throughout proliferation, differentiation, and maturation are limited. Notably, only two publications utilized decellularized adipose tissue (DAT) as a scaffold material for the culture of hASC [8,31]. DAT most closely mimics the native ECM environment and has previously been shown to naturally induce adipogenic differentiation and support hASC proliferation [8].

Cheung et al. found that incorporation of DAT into methacrylated glycol chitosan (MGC) and methacrylated chondroitin sulphate (MCS) hydrogels enhanced long-term viability and hASC adipogenesis based on GPDH enzyme activity, adipogenic gene expression, and intracellular lipid accumulation. Cells seeded on DAT hydrogel scaffolds exhibited better cell retention than culture on a pure DAT scaffold. Additionally, hydrogel choice significantly impacted hASC viability and adipogenesis [31]. These results suggest the need for more studies on the impact of materials on hASC viability and adipogenesis for tissue engineering applications.

Mohiuddin et al. analyzed the effect of seeding hASC on DAT hydrogels in terms of hASC proliferation and differentiation as well as hydrogel microstructure. Their results suggested that the DAT hydrogel could support the attachment, proliferation, and adipogenic differentiation of hASC. Adipogenic differentiation was analyzed by adipogenic gene expression and BODIPY staining. Additionally, DAT hydrogels cultured with hASC in adipogenic media had a lower mean pore size and a higher number of fiber intersections compared to unseeded hydrogels. However, this change in hydrogel structure was less significant than that of constructs cultured in osteogenic media [26]. These findings further confirm the potential use of DAT hydrogels as a scaffold material for hASC throughout adipogenesis. More research needs to be conducted to identify scaffold materials and material properties that best support the proliferation, differentiation, and maturation of hASC in vitro to engineer in vivo-like adipose tissue constructs.

Louis et al. created vascularized adipose tissue constructs by bioprinting a mixture of mature human adipocytes, hASC, human umbilical endothelial cells (HUVEC), collagen microfibers, and fibrinogen into a supporting bath. The constructs remained viable and demonstrated vascularization with capillary structures surrounding mature adipocytes after seven days of culture in vitro [11].

Three-dimensional scaffold culture is typically done in static conditions wherein media is replaced every few days of culture [11,24]. In addition to being labor intensive with a high risk of contamination, static culture systems typically cultivate non-physiological mass transport conditions characterized by heterogeneous nutrient delivery to cells throughout the scaffold. Finally, static culture does not accurately recapitulate interstitial flow through adipose tissue in vivo [11]. Gugerell et al. assessed hASC seeded on P(LLG) scaffolds and in gelatin hydrogels subjected to static and perfusion culture at a flow rate of 0.3 mL/min. Culture in a perfusion bioreactor led to better cell viability after nine days compared to static culture. However, static culture supported enhanced adipogenesis compared to perfusion culture [48,132]. More research needs to be done on the effects of media perfusion and bioreactor geometry on hASC adipogenic differentiation in vitro in order to identify the most effective culture methods for recapitulating nutrient transport through adipose tissue.

#### 3.3.2. Scaffold-Free Culture

Scaffold design for adipose tissue engineering can be challenging due to the significant increase in adipocyte size throughout maturation. Many scaffolds are unable to adapt their pore size, which can restrict adipocyte volume expansion. Therefore, scaffold-free culture approaches leading to the formation of 3D spheroids have been studied extensively. There are four main scaffold-free culture mechanisms: low-attachment culture, hanging drop, magnetic levitation, and dynamic culture conditions [132]. Low-attachment culture involves the formation of spheroids via suspension of cells over a low-attachment culture plate. Hanging drop culture allows for the gravity-mediated formation of spheroids. In magnetic levitation culture, cells are mixed with magnetic particles and subjected to a magnet throughout culture. Finally, dynamic culture conditions include spheroid formation in a spinning flask or microgravity bioreactor [30].

Kapur et al. found that hASC spheroids formed using a hanging drop method followed by adherent or ultra-low attachment culture spontaneously generate an extracellular matrix as evident by positive staining for type I and type III collagen. hASC survived for up to six months in ultra-low attachment culture and were able to produce new, replication-competent cells when moved to adherent culture. Additionally, spheroids demonstrated adipogenic differentiation potential while cultured in ultra-low attachment culture and exposed to adipogenic induction media [10].

Fitzgerald et al. compared three different culture methods: ultra-low attachment static culture, ultra-low attachment dynamic culture, and elastin-like polypeptide–polyethyleneimine (ELP–PEI) coated surfaces. They assessed spheroid size and number of spheroids through time-lapse microscopy and adipogenic differentiation by measuring triglyceride content. They found that ultra-low attachment static and dynamic culture led to a reduction in number of spheroids due to spheroid merging and ultimately the formation of larger spheroids. PEI molecular weight was shown to impact cell retention and spheroid size as lower molecular weight PEI (800 g/mol) exhibited better retention and larger spheroid size than higher molecular weight PEI (25,000 g/mol). Additionally, triglyceride accumulation on ELP–PEI coatings was equal to or higher than that in ultra-low attachment static and suspension culture [48].

Zhang et al. assessed the effects of hASC spheroid formation in a microgravity bioreactor on stemness properties and differentiation potential. Spheroids exhibited increased stemness properties, including increased proliferation and colony forming efficiency, compared to monolayer culture. Additionally, spheroids exhibited increased adipogenic differentiation capabilities as demonstrated by an upregulation of adipogenic marker genes (PPAR-γ and LPL) and ORO staining [38].

Labusca et al. used proprietary magnetic nanoparticles and magnetic field levitation to form hASC spheroids. Spheroids exhibited improved viability and proliferation compared to non-levitated spheroids and 2D culture. Additionally, hASC experienced increased adipogenesis when cultured in spheroids compared to 2D; however, magnetic field levitation was associated with a smaller increase in adipogenesis compared to non-levitated spheroids [20].

Results from these studies indicate the potential use of scaffold-free hASC culture methods for adipose tissue engineering applications. While 3D hASC spheroid culture has demonstrated increased adipogenic differentiation potential and improved maintenance of stemness properties, more research needs to be conducted to determine the best scaffold-free culture methods for engineering adipose tissue. Results from this systematic review indicate that studies on 3D culture methods in the field primarily use “snapshot” assays (adipogenic gene expression, etc.) rather than functional assays (lipolysis, glucose uptake, etc.) to assess hASC adipogenesis in vitro. Further research is needed to evaluate the impact of 3D culture methods on hASC functionality to engineer adipose tissue constructs that are more physiologically relevant.

### 3.4. Microphysiological Systems

**Figure 6:**
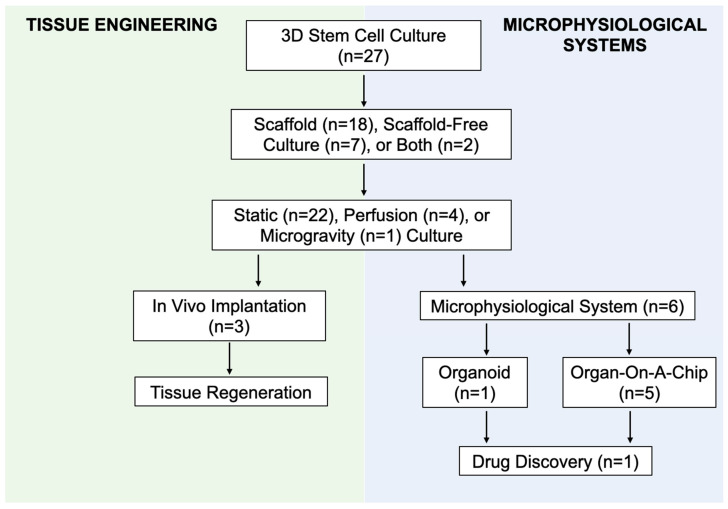
Flow diagram demonstrating the intersection between culture methods for adipose tissue engineering and microphysiological systems (MPS).

Scaffold and scaffold-free 3D culture techniques have been employed to develop microphysiological models of adipose tissue. Adipose tissue MPS aim to recapitulate the functionality of adipose tissue as an organ system in vitro with the ultimate goal to reduce, refine, and replace animal models during the drug discovery process for human diseases. Out of the relevant articles included in this review, six articles (22.2%) self-reported the use of an adipose tissue MPS in their research. One article reported the use of an adipose tissue organoid. The remaining five articles reported the use of an adipose tissue “organ-on-a-chip” (Figure 6).

Tseng et al. published a methods paper about their successful development of an “adiposphere” via magnetic levitation of human SVF, preadipocytes and endothelial cells, and hASC [17]. This is an example of a scaffold-free culture mechanism used to create an MPS, specifically, an organoid. Few studies have been done on the development of adipose tissue organoids and assessment of their potential for aiding drug discovery and personalized medicine.

Paek et al. created a vascularized adipose tissue microphysiological system by coculturing hASC and human adipose microvascular endothelial cells (hAMECs) in a fibrin hydrogel scaffold. The hAMECs underwent vasculogenesis and formed a network of endothelial tubes throughout the scaffold. The presence of the vasculature increased the rate of adipogenesis allowing for the formation of twice as many lipid droplets in 28 days compared to a non-vascularized model. Furthermore, cells were cultured in the MPS for 40 days leading to the robust maturation and growth of adipocytes to form a densely packed tissue construct [24].

Yang et al. created a microfluidic device to study the effects of flow rate on hASC adipogenic differentiation. The device is composed of five round, 3D culture chambers filled with a hASC-laden fibrin hydrogel scaffold interconnected by two fluidic channels. By administering media at a volumetric flow rate of 1, 4, and 10 µL/hr, average cellular shear stress was estimated to be 0.79 mPa, 3.13 mPa, and 7.86 mPa, respectively. Despite the fact that all three average shear stresses are within physiologic limits (less than 10 mPa), several adipogenic markers were downregulated in response even the smallest amount of shear stress. This suggests that even physiological levels of interstitial shear stress can have inhibitory effects on hASC adipogenesis in vitro [4]. More studies need to be conducted on the effects of media flow rate and MPS geometry on hASC adipogenesis. Solidifying an effective method of modeling interstitial flow throughout adipose tissue without compromising hASC adipogenic potential will allow for the development of a model that better recapitulates in vivo mass transport and supports long-term cell viability.

Bender et al. used human SVF and a human blood product-derived biological scaffold (ObaGel™) to create a “fat-on-a-chip” construct. These 3D structures demonstrated increased adipogenesis based on number and size of lipid vacuoles. Additionally, SVF cultured in ObaGel demonstrated increased glucose uptake, leptin secretion, and lipolysis in response to isoproterenol, metformin, and compound C compared to 2D culture [10,48].

Several reports identified in this systematic review discuss the use of an adipose tissue MPS. Despite significant findings that hASC culture in 3D cellular aggregates (spheroids) experience increased adipogenesis likely due to the replication of in vivo adipocyte morphology, there is a lack of research on the development of self-assembled adipose tissue organoids [4]. Additionally, it is possible that the lack of both organoid and organ-on-a-chip models identified in this review is, in part, due to inconsistency in the classification of MPS. Therefore, there is a need for the standardization of MPS definitions and classification criteria within the field.

Articles that self-identified as using an MPS tended to use more functional assays for characterizing their in vitro adipose tissue constructs compared to articles that did not identify as using an MPS. This is likely due to the focus of MPS development for drug discovery applications which necessitates the formation and, therefore, validation of physiologically relevant adipose tissue. Even so, there is a general lack of focus on functional assays within the published literature. Additionally, only one article in this review performed drug testing on a construct [4]. Therefore, there is a need for increased focus on assessing the functionality of in vitro adipose tissue constructs derived from hASC and human SVF to understand the impact of culture conditions on the development of physiologically relevant adipose tissue for drug discovery applications.

### 3.5. Limitations of Current Analysis

Despite the systematic approach utilized in the current meta-analysis, there are several limitations to the analysis in this systematic review. The keywords used in the database search did not capture all relevant articles, as evident by the inclusion of several related articles identified only through independent search efforts. It is possible that this was amplified through the use of only a single literature database (PubMed). Additionally, we limited our search to articles that used either adipose-derived stem cells or stromal vascular fraction from human sources. This potentially limited the methods that we identified by not including studies that used animal-derived sources. Additionally, with a narrow focus on hASC and SVF culture methods, we were not able to capture related studies that may have used different cell types such as mature adipocytes or explanted adipose tissue constructs.

### 3.6. Identifying Future Directions Using MPS Models to Evaluate Human Adipose Biology

Significant advances have been made in developing physiologically relevant MPS models of adipose tissue using hASC and SVF cells. However, there are several gaps in the published literature that were identified in this systematic review.

First, there is no standardized cocktail of adipogenic factors that are used to routinely induce either beige/brown or white adipose differentiation. In the absence of such a fundamental standardized growth medium, there is likely to be considerable variability in the adipogenic process across laboratories and studies. Despite the widespread use of ECM-derived biomaterial scaffolds for culturing hASC, there are few reports on cell-material interactions throughout proliferation, adipogenesis, and maturation. Among articles studying scaffold-free culture methods, there are few reports on the impact of culture type on adipogenic potential. Additionally, no articles identified in this review used functional assays to assess the impact of 3D culture methods on the creation of a physiologically functional in vitro tissue construct. Therefore, there is a need for more studies to assess the impact of culture environment on the development of physiologically relevant adipose tissue constructs.

Similarly, very few articles reported the use of perfusion culture to mimic interstitial flow in adipose tissue. Articles using perfusion culture reported decreased adipogenic potential compared to static culture [17,26,45]. There is a need for more research on the impact of flow rate and bioreactor geometry to develop culture conditions that support maximum cell viability by mimicking in vivo mass transport without compromising hASC adipogenic potential.

Despite the fact that white adipose tissue is dependent on vascularization for the transport of nutrients, oxygen, and other soluble factors to maintain homeostasis, only three articles identified in this review focused on engineering vascularized adipose tissue constructs [17]. Notably, Paek et al. found that the vascularization of the tissue construct was associated with increased adipogenesis compared to a non-vascularized construct [4]. However, no articles included in this review used functional assays to assess the impact of vascularization on the functionality of adipose tissue constructs in vitro. This is a critical next step to the development of physiologically accurate models of human adipose tissue. Finally, there were a limited number of studies that used MPS models. Only one article studied the impact of drug application on a tissue construct [4]. Additionally, there were no articles that modeled adipose tissue disease conditions. The future of MPS modeling for effective drug screening relies upon advancements in these areas.

**Figure 7:**
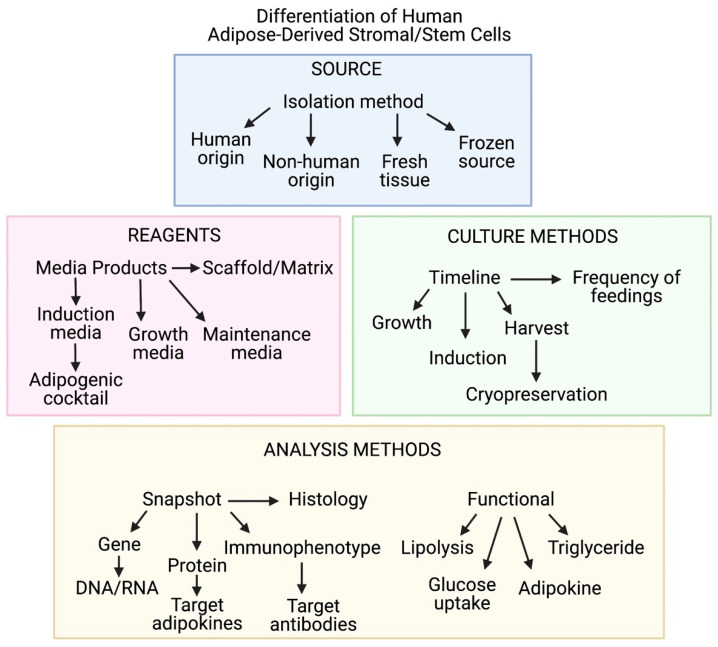
Diagram demonstrating critical variables associated with in vitro modeling of human adipose tissue. Imaged created with BioRender.com.

According to the FDA, predictive tools such as engineered tissue constructs are critical to the goal of reducing, refining, and/or replacing animal testing. Standardization of variables such as reagents, culture methods, and analysis methods (summarized in Figure 7) used for adipose tissue engineering will propel the field towards models with enhanced physiological relevance. This will support the FDA’s mission to further predictive toxicology and enhance safety when bringing new medical products to market [6].

In summary, there remains a need for an increased focus on the assessment of in vitro functionality within the field of adipose tissue engineering for both regenerative medicine and MPS models. Advancements in 3D culture methods for the generation of physiologically relevant adipose tissue constructs will synergistically support the discovery of novel methods for soft tissue regeneration and models for drug discovery.

## Figures and Tables

**Table 1:** Number of articles identified in PubMed using seven subsets of search terms.

Search Terms	Articles Identified
Adipose, three dimensional, spheroid, human	77
Adipose, three dimensional, microphysiological system, human	4
Adipose, three dimensional, xenografts, human	10
Adipose, three dimensional, iPSC, human	9
Adipose, three dimensional, hydrogel scaffold, human	60
Adipose, three dimensional, organoids, human	10
Adipose, three dimensional, induced pluripotent stem cell, human	9

**Table 2:** Adipogenic medium components used in custom-made media. Results reported as the percentage of papers that used a given adipogenic medium component.

Adipogenic Medium Components	Percent Usage in Custom-Made Medium
Indomethacin	60%
Isobutylmethylxanthine (IBMX)	86.7%
Rosiglitazone	0%
Dexamethasone	93.3%
Insulin	93.3%
Biotin	13.3%
Pantothenate	13.3%
Other	26.7%

**Table 3:** hASC biomarkers evaluated using flow cytometry.

Report	Positive Markers Identified	Negative Markers Identified
Clevenger et al.	CD44, CD90, CD105	CD45, CD31
Keck et al.	CD90, CD105, CD44	CD45
Mohiuddin et al.	CD73, CD90, CD105	CD3, CD14, CD31, CD45
Shen et al.	CD90, CD105	
Bender et al.	CD29, CD105, CD34, CD73, CD90	CD45

**Table 4:** Most common synthetic and biologically-derived scaffold materials reported in the literature. Results reflect the number of articles in which a specific material is used as a component in a scaffold.

Biologically-Derived Scaffold Materials	Number of Appearances in the Literature	Appearances in the Literature
Collagen	5	Labriola et al., 2018; Newman et al., 2020; O’Donnell et al., 2020; Paek et al., 2019; Vinson et al., 2017
Gelatin	4	Gugerell et al., 2015; Lau et al., 2018; O’Donnell et al., 2020; Vinson et al., 2017
Fibrin	3	Keck et al., 2019; Paek et al., 2019; Yang et al., 2021
Decellularized Adipose Tissue	2	Cheung et al., 2014; Mohiuddin et al., 2019
Alginate	2	Lee et al., 2014; Vinson et al., 2017
**Synthetic Scaffold Materials**		
Polyethylene Glycol	3	Clevenger et al., 2016; Lee et al., 2014; Reid et al., 2013
Methacrylate	3	Cheung et al., 2014; Gugerell et al., 2015; O’Donnell et al., 2020
